# Artesunate ameliorates cigarette smoke-induced airway remodelling via PPAR-γ/TGF-β1/Smad2/3 signalling pathway

**DOI:** 10.1186/s12931-021-01687-y

**Published:** 2021-03-23

**Authors:** Kunming Pan, Juanjuan Lu, Yun Song

**Affiliations:** 1 Department of Pharmacy, Zhongshan Hospital Fudan University, Shanghai, 200032 China; 2 Department of Pharmacology, Shanghai Jiao Tong University School of Medicine, Shanghai, 200025 China; 3 Department of Pharmacy, Huashan Hospital Fudan University, Shanghai, 200040 China

**Keywords:** Artesunate, COPD, Airway remodelling, Cigarette smoke, PPAR-γ

## Abstract

**Background:**

Airway remodelling is the major pathological feature of chronic obstructive pulmonary disease (COPD), and leads to poorly reversible airway obstruction. Current pharmacological interventions are ineffective in controlling airway remodelling. In the present study, we investigated the potential role of artesunate in preventing and treating airway remodelling and the underlying molecular mechanisms in vitro and in vivo.

**Methods:**

A COPD rat model was established by cigarette smoke (CS) exposure. After 12 weeks of artesunate treatment, pathological changes in the lung tissues of COPD rats were examined by ELISA and histochemical and immunohistochemical staining. A lung functional experiment was also carried out to elucidate the effects of artesunate. Human bronchial smooth muscle (HBSM) cells were used to clarify the underlying molecular mechanisms.

**Results:**

Artesunate treatment inhibited CS-induced airway inflammation and oxidative stress in a dose-dependent manner and significantly reduced airway remodelling by inhibiting α-smooth muscle actin (α-SMA) and cyclin D1 expression. PPAR-γ was upregulated and TGF-β1/Smad2/3 signalling was inactivated by artesunate treatment in vivo and in vitro. Furthermore, PPAR-γ knockdown by siRNA transfection abolished artesunate-mediated inhibition of HBSM cell proliferation by activiting the TGF-β1/Smad2/3 signalling pathway and downregulating the expression of α-SMA and cyclin D1 in HBSM cells.

**Conclusions:**

These findings suggest that artesunate could be used to treat airway remodelling by regulating PPAR-γ/TGF-β1/Smad signalling in the context of COPD.

**Supplementary Information:**

The online version contains supplementary material available at 10.1186/s12931-021-01687-y.

## Introduction

Airway remodelling is a major pathological abnormality in chronic obstructive pulmonary disease (COPD) [1]. Oxidative stress induced by environmental factors, including exposure to cigarette smoke (CS), has been shown to induce airway hyperreactivity and airway remodelling, and these effects involve airway smooth muscle [2–4]. Airway remodelling is also caused by the upregulation of α-smooth muscle actin (α-SMA) and Cyclin D1, which are critical regulators of cell proliferation and cell cycle progression [5].

Ligand-activated transcription factor peroxisome proliferator-activated receptor γ (PPAR-γ), a member of the nuclear hormone receptor superfamily, is frequently expressed in human airway smooth muscle cells [6]. Increasing evidence has revealed that PPAR-γ agonists can inhibit the proliferation of human airway smooth muscle cells, and reduce inflammatory cell infiltration and airway remodelling by activating PPAR-γ in the context of COPD [7–9]. In addition, PPAR-γ was also reported to inhibit the activation of TGF-β1-Smad2/3 pathway, which plays a vital role in the progression of epithelial–mesenchymal transition (EMT) and airway remodelling [10, 11]. Therefore, targeting PPAR-γ signalling could represent an effective strategy for the preventingand treating airway remodelling in COPD.

Current clinical pharmacological therapies, including corticosteroids and bronchodilators, are able to reduce exacerbations and improve symptoms but cannot suppress the development and progression of COPD [12]. Drug repurposing is one such strategy for treating COPD. The repurposing of ‘old’ drugs is gradually becoming an attractive option because it involves the use of less risky compounds with potentially lower overall development costs and shorter development timelines [13]. Artesunate is a semisynthetic derivative of the Chinese herb *Artemisia* *annua* L., which is commonly used as an antimalarial agent [14]. In addition, artesunate has been shown to possess anti-inflammatory and antioxidant activity [15, 16]. A recent report demonstrated that artesunate ameliorated oxidative lung damage in experimental allergic asthma and attenuated CS-induced lung damage and emphysema in mice [17, 18]. Artesunate could also inhibit the proliferation of primary human cultured airway smooth muscle cells [19, 20] and improve bleomycin-induced pulmonary fibrosis pathology in rats by inhibiting TGF-β1–Smad3 activation [21]. However, the effects of artesunate on airway remodelling and the underlying mechanism(s) remain to be further explored.

In the present study, we hypothesized that airway inflammation and airway remodelling, which are already accompanied by clearly abnormal lung function that is ameliorated by artesunate, are closely associated with the PPAR-γ/TGF-β1/Smad signalling pathway. Using human bronchial smooth muscle (HBSM) cells and an experimental rat model of COPD, the present study described the protective effects of artesunate and the underlying mechanism(s). The present study provides new uses for the old drug artesunate and the potential mechanisms by which artesunate affects airway inflammation and remodelling in COPD.

## Materials and methods

### Rat model of COPD

Thirty Sprague-Dawley (SD) rats (8 weeks, 230 ± 25 g, half male and half female) were purchased from Shanghai Laboratory Animal Company (Shanghai, China). All rats were housed in a room temperature of 25 °C and in a light–dark cycle of 12:12 h with free access to diet and water ad libitum. All animal experiments were approved by the Experimental Animal Ethics Committee of Fudan University and performed in accordance with the guidelines for the care and use of laboratory animals set by Fudan University (Shanghai, China).

30 rats were randomly divided into five groups (6 rats/group) as follows: Control group, CS group, CS + 25 mg/kg Artusenate group, CS + 50 mg/kg Artusenate group, CS + 100 mg/kg Artusenate group. The CS-induced COPD rat model was established as our previous report [22]. Rats were exposed to cigarette smoke from four cigarettes (Double Happiness, Shanghai) burning simultaneously and each exposure lasted 75 min consuming 48 cigarettes totally. The smoke exposure was performed twice per day and 5 days per week for 12 weeks by using a custom-designed and purpose-built nose-only, directed flow inhalation and smoke-exposure system (handmade) housed in a fume and laminar flow hood. Artesunate (25, 50, 100 mg/kg) was administered via intraperitoneal injection 1 h before the first exposure of a day. Control group was treated with normal saline. The rats were anesthetized with 40 mg/kg intraperitoneal sodium pentobarbital for sample collection and further analysis after 12 weeks of smoke exposure.

### Lung function test

Lung function was measured as we previously described [22]. After anaesthesia, trachea cannula was inserted at the throat to examine lung function by PowerLab 8sp Life Analysis System (AD Instrument, Australia), including peak inspiratory flow (PIF), peak expiratory flow (PEF), airway inside pressure (IP) and airway pressure maximum rising slope (IP-slope).

### Bronchoalveolar lavage fluid (BALF)

After rats were anesthetized by injecting sodium pentobarbital, tracheotomy was performed and a cannula was inserted into the trachea. BALF was collected from the right lungs through three lavages of 1 ml phosphate-buffered saline (PBS). Extracted BALF was immediately centrifuged at 1000 rpm for 5 min at 4 °C, and then used for further assays.

### Airway smooth muscle isometric tension assay

The airway isometric contraction assay was performed as described previously [5]. To measure airway smooth muscle isometric tension of rats, the main bronchus was rapidly separated from surrounding connective tissue after the extraction of BALF. The bronchial rings were fixed on the stainless-steel hooks in a 37 °C bath of modified Kreb’s solution (composition in mM: NaCl 118; KCl 4.7; MgSO_4_ 1.2; KH_2_PO_4_ 1.2; NaHCO_3_ 25; CaCl_2_ 2.5; glucose 11, EDTA·Na_2_ 0.5) and then continuously bubbled with 95% O_2_ and 5% CO_2_. The bronchial rings were connected vertically to a force–displacement transducer under a resting tension of 500 mg. After the separated bronchial rings were washed every 15 min for 1 h, the values of isometric tension were recorded by PowerLab 8sp Life Analysis System (AD Instruments, Sydney, Australia) to generate the cumulative concentration-response curves for carbachol (CCh). The values of isometric tension were expressed as force.

### Histology and immunohistochemistry

After bronchoalveolar lavage, the right lungs were immersed in 4% paraformaldehyde for 24 h. After fixation, paraffin embedding and section, H&E and Masson stainnig were performed for inflammation and fibrosis evaluation, respectively. Immunohistochemistry staining of α-SMA and cyclin D1 (CST, Cell Signalling Technology, Beverly, MA, USA) was performed for remodling evaluation. Quantitative analysis was performed by Image-Pro plus 6.0.

### Cell culture, cell transfection and cigarette smoke extract (CSE) preparation

The human bronchial smooth muscle (HBSM) cells were purchased from the ScienCell Research Laboratories (San Diego, California, USA). Cells were cultured in Smooth Muscle Cell Medium (ScienCell, San Diego, California, USA) and performed for experiments at passage 2–4 with no mycoplasma contamination. HBSM cells were divided into five groups: Control group, CSE group, CSE + 1 µM Artusenate group, CSE + 10 µM Artusenate group, CSE + 100 µM Artusenate group. HBSM cells were pre-incubated with or without artesunate (1, 10, 100 μM; Sigma-Aldrich, USA), rosiglitazone (RGZ, 100 μM, Sigma-Aldrich, USA) or GW9662 (10 μM, Sigma-Aldrich, USA) for 1 h, and subsequently co-incubated with 2.5% CSE for 24 h.

The small interfering RNAs (siRNAs) were synthesized by Genema (Shanghai, China) for the PPAR-γ knockdown assay. The sequences of si-PPAR-γ are as follows: 5′-TGGAAATGTGATACGCAAAAT-3′. The siRNA transfection was conducted with Lipofectamine 3000 (Invitrogen, Waltham, MA, USA) according to the manufacturer’s protocol. The knockdown experiments included five groups as follows: Negative control group, PPAR-γ siRNA group, PPAR-γ siRNA + CSE group, CSE + Artusenate group, PPAR-γ siRNA + CSE + Artusenate group.

CSE was prepared as our previous reports [22]. The CSE was prepared by combusting one cigarette (Double happiness, Chinese, the amount of tar was 12 mg), using a pump and passing the smoke through 10 ml of FBS-free culture medium at a rate of 5 min/cigarette. The resulting solution was adjusted to pH 7.4 with 1 mol/l of concentrated NaOH and filtered through a 0.22 μm filter. The obtained solution was referred to as 100% strength and diluted to the desired concentrations with culture medium.

### Measurement of indicated factors and cell proliferation

After above indicated treatment, BALF or cell supernatants were collected to examine TGF-β1, IL-8, TNF-α, IL-6 and ICAM-1 level using an enzyme-linked immunosorbent assay (ELISA, Elabscience Biotechnology, China). Reactive oxygen species (ROS) production was measured by H2DCFDA reagent (Sigma, St. Louis, MO, USA) using flow cytometry. Cell lysate was used to measure intracellular GSH with a commercial assay kits (Beyotime, Jiangsu, China) as manufacturer’s instructions. The cell proliferation was measured by Cell Counting Kit (CCK)-8 assay and BrdU cell proliferation assay kit (CST, Beverly, MA, USA) according to manufacturer’s protocols.

### Western blot analysis

After serum deprivation for 24 h, HBSM cells were cultured in DMEM with CSE stimulation in the presence or absence of artesunate. α-SMA, Cyclin D1 and GAPDH expression were measured after 24 h. Frozen lung tissues were homogenized in RIPA lysate using ultra-sonic oscillation, total protein extracts were separated by 10% SDS-PAGE. Immunoblots were probed with anti-α-SMA, anti-cyclin D1, anti-PPAR-γ, anti-p-Smad2, anti-p-Smad3, anti-Smad2, anti-Smad3, anti-GAPDH and anti-β-actin (CST), followed by horseradish peroxidase-conjugated secondary antibody (Abcam). Protein bands were visualized with Biorad System (USA), β-actin and GAPDH were used as internal controls for total protein extracts, respectively. Band intensity was quantitated using Image J software.

### Statistical analyses

All the experimental data are presented as the means ± SEM and analyzed by Prism version 6.0 (GraphPad Software, San Diego, USA). The *t*-test was performed to measure the differences between the two groups and one-way analysis of variance (ANOVA) followed by a Dunnett’s test was performed to compare the differences among three or more groups. *P*-values < 0.05 were considered statistical significance.

## Results

### Role of artesunate in reducing airway inflammation and oxidative stress in the lungs of CS-exposed rats

**Fig. 1:**
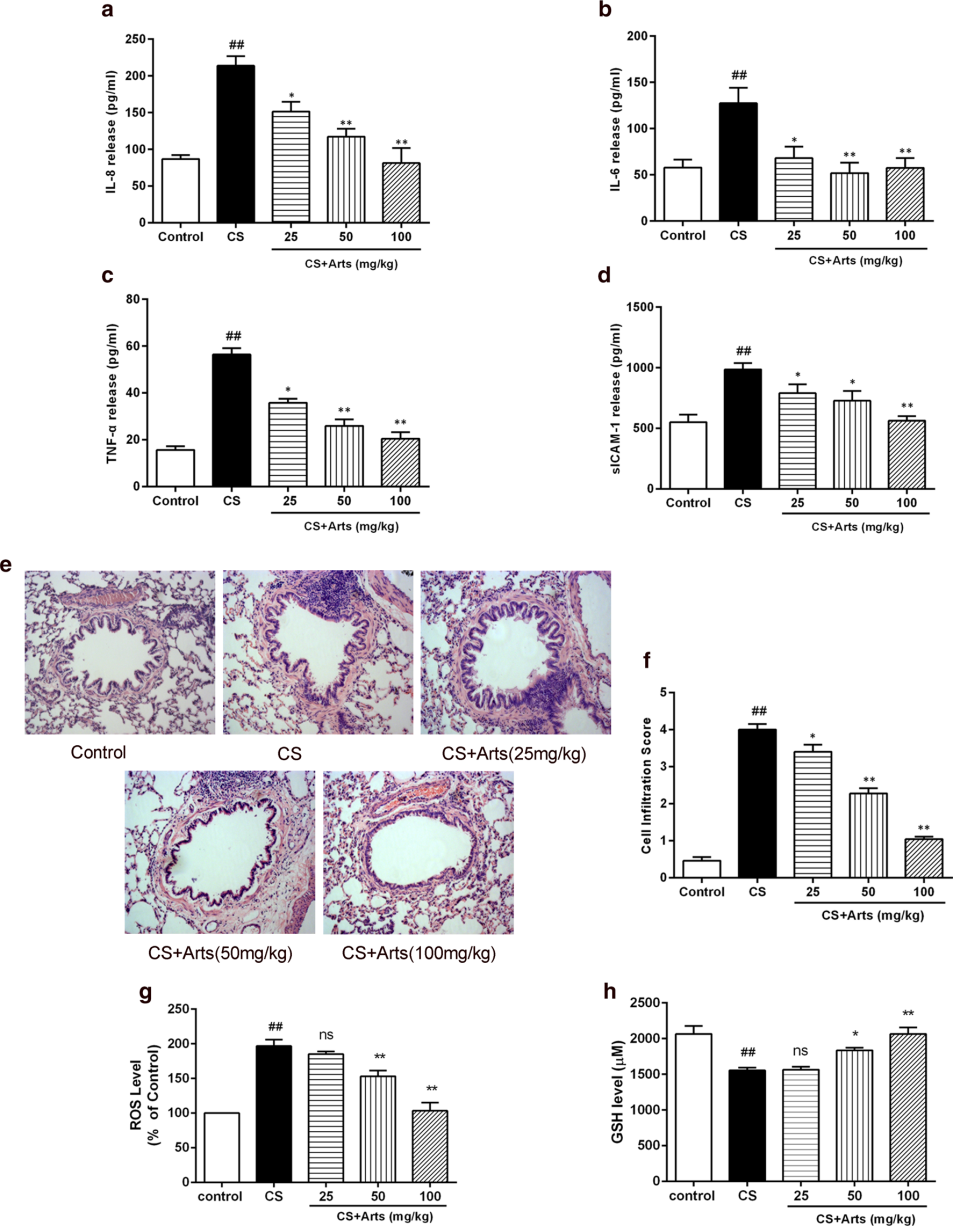
Artesunate reduced CS-induced airway inflammation and oxidative stress in rats. After CS exposure for 12 weeks, rats were euthanized and lungs and bronchoalveolar lavage fluid (BALF) were collected. **a**–**d** The concentrations of IL-6, IL-8, TNF-α, and ICAM-1 in the BALF were measured by ELISA. **e**, **f** H&E staining was performed for assessment of the inflammatory infiltration. **g**, **h** The ROS and GSH concentration in BALF were measured by kits. Data were expressed as mean ± SEM, n = 6. ^##^P < 0.01 compared to control group using unpaired *t*-test; *P < 0.05, **P < 0.01 and ns means no significant difference compared to CS group using one-way ANOVA with Dunnett test for selected pairs. *Arts* Artesunate

IL-6, IL-8, TNF-α, and ICAM-1, which amplify the inflammatory process and induce airway structural changes were examined in BALF after 12 weeks of CS exposure. The levels of IL-6, IL-8, TNF-α, and ICAM-1 in BALF were significantly higher in the CS-exposed group than in the control group (Fig. 1a–d). The increase in IL-6, IL-8, TNF-α, and ICAM-1 was dose-dependently attenuated by artesunate treatment (Fig. 1a–d). Consistently, artesunate treatment also markedly attenuated inflammatory infiltration, which was significantly increased in the lungs of CS-exposed rats compared to control rats (Fig. 1e, f).

Oxidative stress induced by CS exposure also largely correlates with the process of airway remodelling [23]. As shown in Fig. 1g, CS significantly increased ROS levels in BALF, and this effect was reversed by artesunate treatment. Furthermore, the decreased GSH level induced by CS exposure was also significantly reversed by artesunate treatment (Fig. 1h). Taken together, these findings demonstrated that artesunate could protect against airway inflammation and oxidative stress in a CS exposure rat model and suggested a potential role of artesunate in the prevention and treatment of airway remodelling.

### Role of artesunate in attenuating CS-induced airway remodelling in rats

**Fig. 2:**
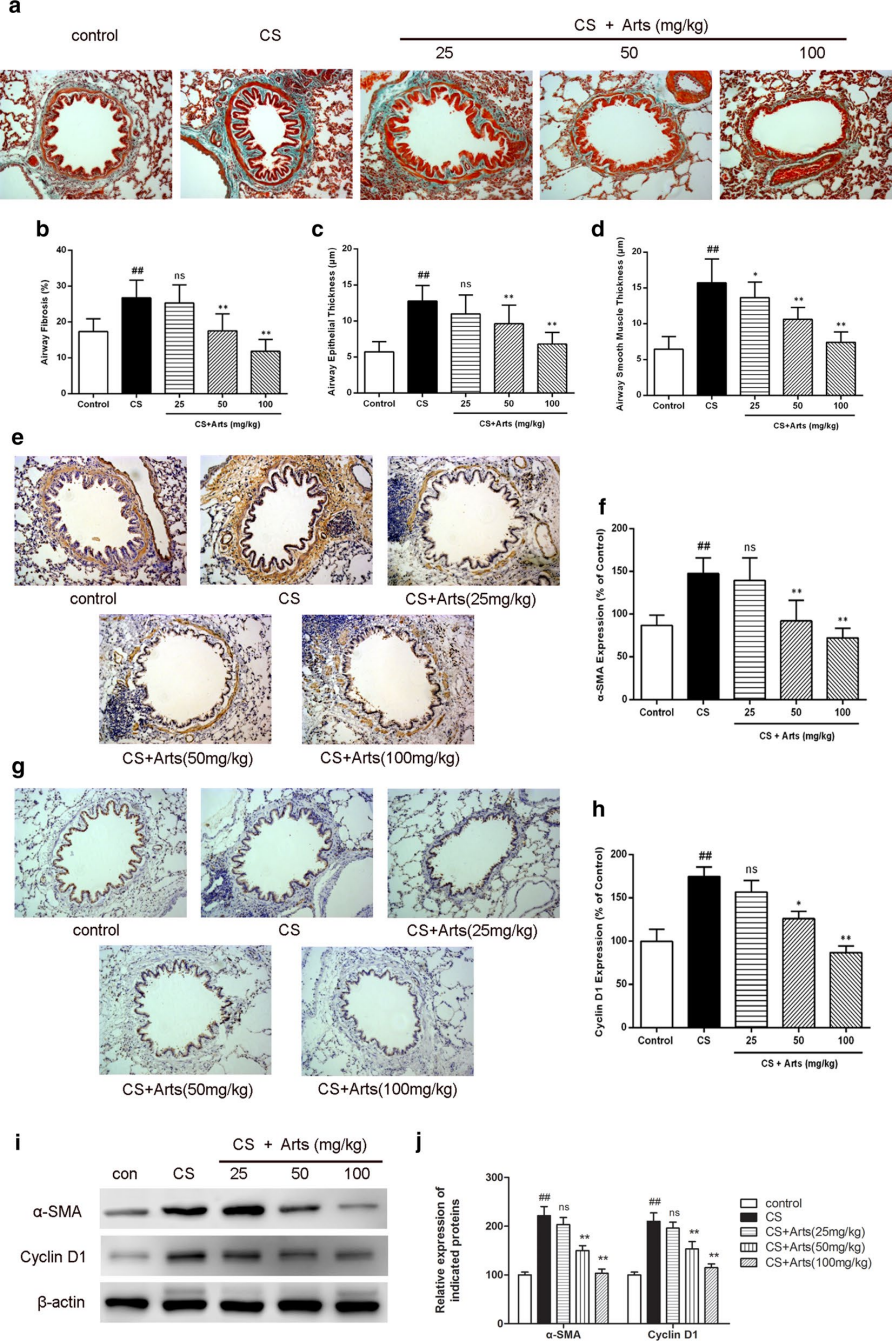
Artesunate attenuated CS-induced airway remodelling in rats. **a** Masson’s trichrome staining was performed to determine airway histopathological changes. **b**–**d** The airway fibrosis, airway epithelial thickness and airway smooth muscle thickness were quantified by Image J. **e**–**h** Immunohistochemical staining were performed to determine α-SMA and cyclin D1 expressions in the lungs. **i**, **j** The proteins levels of α-SMA and cyclin D1 in the lungs were analyzed by Western blot. Data were expressed as mean ± SEM, n = 6, ^##^P < 0.01 compared to control group using unpaired *t*-test; *P < 0.05, **P < 0.01 and ns means no significant difference compared to CS group using one-way ANOVA with Dunnett test for selected pairs. *Arts* Artesunate

Airway remodelling, which is generally accepted, is closely associated with persistent chronic inflammation and oxidative stress [24]. An clear airway remodelling phenotype characterized by fibrosis and epithelial and smooth muscle thickness, was observed after 12 weeks of CS exposure, and these CS-induced changes were significantly reversed by artesunate in a dose-dependent manner (Fig. 2a–d). α-smooth muscle actin (α-SMA) and cyclin D1 are key mediators of airway remodelling [5]. In the CS exposure groups, the expression of α-SMA and cyclin D1 in the lung was dramatically upregulated and reversed by artesunate treatment (50 mg/kg and 100 mg/kg), as measured by IHC (Fig. 2e–h) and western blotting (Fig. 2i, j). These results suggest that airway remodelling following CS-exposure in rats could be reversed by artesunate treatment. In addition, the results of correlation analysises showed that cyclin D1 expression in lung tissues was significantly correlated with both airway smooth muscle thickness (P < 0.01, r^2^ = 0.5403) and airway epithelial thickness (P < 0.05, r^2^ = 0.5664) (shown in Additional file 1).

### Role of artesunate in ameliorating lung function and airway hyperresponsiveness in CS-exposed rats

**Fig. 3:**
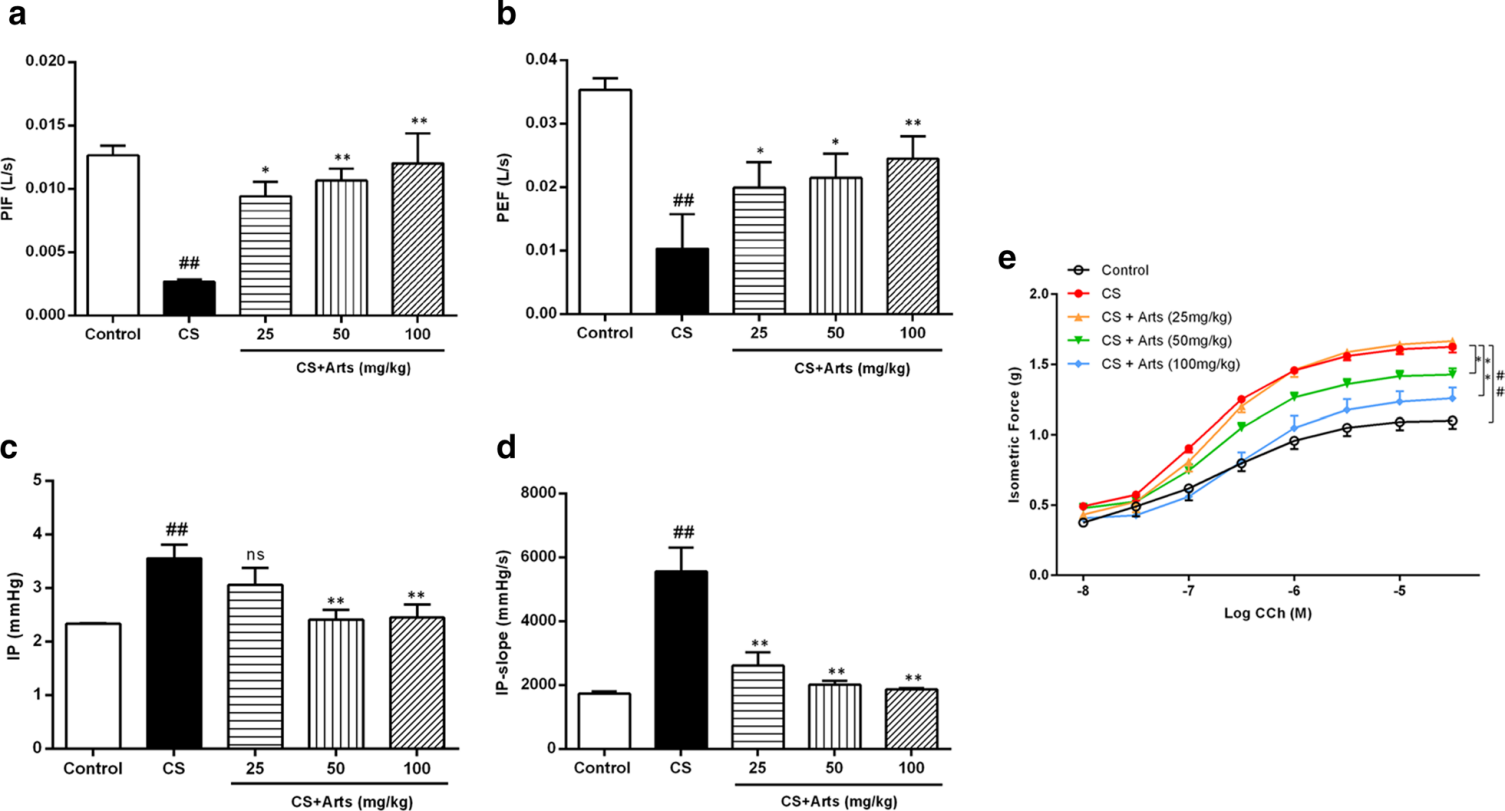
Artesunate restored lung function declining and airway hyperresponsiveness caused by CS. After CS exposure for 12 weeks, the lung function of rats was assessed by using a PowerLab 8sp Life Analysis System, including **a** peak inspiratory flow (PIF), **b** peak expiratory flow (PEF), **c** inspiratory pressure (IP), **d** airway pressure maximum rising slope (IP-slope). **e** isometric force. Data were expressed as mean ± SEM, n = 6, ^##^P < 0.01 compared to control group using unpaired *t*-test; *P < 0.05, **P < 0.01and ns means no significant difference compared to CS group using one-way ANOVA with Dunnett test for selected pairs. *Arts* Artesunate

Airway remodelling contributes to the progressive loss of lung function, and declining lung function caused by persistent airflow obstructions is an important feature of COPD [25]. We next assessed the effects of artesunate on lung function in CS-exposed rats. PIF (Fig. 3a) and PEF (Fig. 3b) significantly decreased after CS exposure for 12 weeks, while IP (Fig. 3c), IP-slope (Fig. 3d) and isometric force (Fig. 3e) significantly increased compared with those of rats in the control group (P < 0.01). Importantly, artesunate treatment improved CS-induced pathological alterations in lung functions, including PIF, PEF, IP and IP-slope (Fig. 3a–d), as well as isometric force (Fig. 3e) compared to those of CS-exposed rats. These results demonstrated that declining lung function caused by airway remodelling could be ameliorated by artesunate treatment under CS-induced oxidative stress conditions.

### Role of artesunate in inhibiting CSE-induced HBSM cell proliferation

**Fig. 4:**
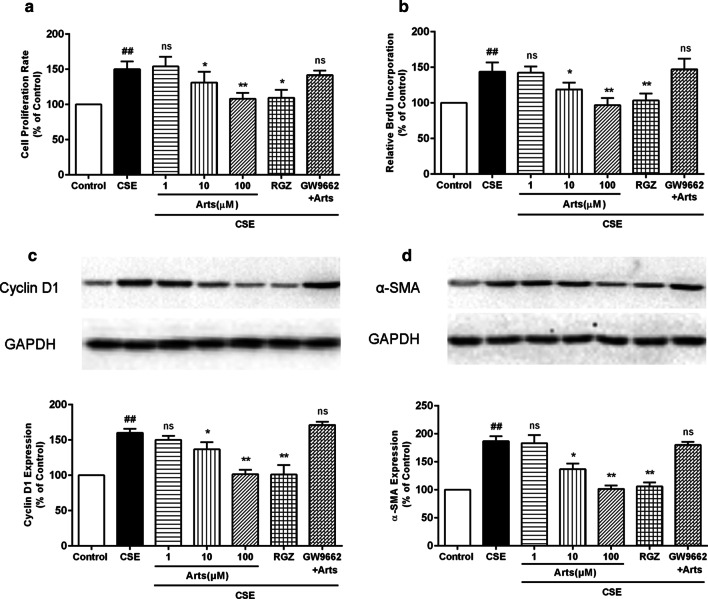
Artesunate inhibited the CSE-induced proliferation in HBSM cells. **a** The cell viability was determined by CCK-8 assay. **b** The cell proliferation was determined by BrdU incorporation assay. The proteins levels of cyclin D1 (**c**) and α-SMA (**d**) in HBSM cells were analyzed by Western blot. Data were expressed as mean ± SEM, n = 3. ^##^P < 0.01 compared to control group using unpaired *t*-test; *P < 0.05, **P < 0.01 and ns means no significant difference compared to CSE group using one-way ANOVA with Dunnett test for selected pairs. *Arts* Artesunate

To further validate the potential role of artesunate in airway remoldling, CCK-8 and BrdU incorporation assays were used to assess the proliferation of HBSM cells. We found that the significantly increased proliferation of HBSM cells triggered by low concentration of CSE (2.5%) was inhibited by treatment with artesunate in a concentration-dependent manner, as indicated by both CCK-8 (Fig. 4a) and BrdU incorporation assays (Fig. 4b). As expected, treatment with artesunate also significantly attenuated CSE-induced cyclin D1 (Fig. 4c) and α-SMA (Fig. 4d) expression as compared to that of CSE alone. These results indicated that artesunate has an inhibitory effect on the proliferation of HBSM cells by attenuating the expression of cyclin D1 and α-SMA. Furthermore, rosiglitazone (RGZ, 100 µM), the PPAR-γ agonist, exerted a similar therapeutic effect to artesunate on CSE-induced HBSM cells, while the addition of PPAR-γ antagonist GW9662 (10 µM) has dramatically reversed the protective effect of artesunate, indicating that PPAR-γ might be the key target of articulate (Fig. 4a–d).

### Role of artesunate in the expression of PPAR-γ and TGF-β1/Smad2/3 activation in both HBSM cells and rat lungs exposed to CS

**Fig. 5:**
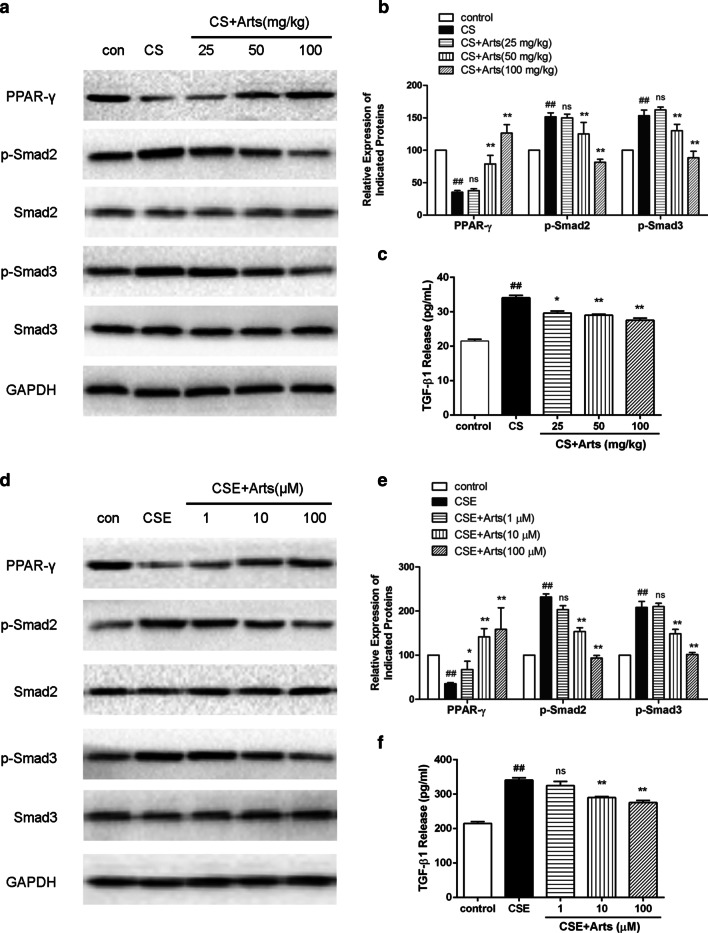
Effects of artesunate on expression of PPAR-γand activation of TGF-β1**/**Smad2/3 signalling in HBSM cells (n = 3) and rat lungs (n = 6). The expressions of PPAR-γ, p-Smad2/3, Smad2/3 and GAPDH in rat lungs (**a**, **b**) and HBSM cells (**d**, **e**) were determined using Western blot. The levels of TGF-β1 in supernatant of BALF of rats (**c**) and HBSM cells (**f**) were determined using ELISA test. Data were expressed as mean ± SEM. ^##^P < 0.01 compared to control group using unpaired *t*-test; *P < 0.05 and, **P < 0.01 and ns means no significant difference compared to CS group using one-way ANOVA with Dunnett test for selected pairs. *Arts* Artesunate

Next, we validated whether the PPAR-γ pathway was involved in the protective effects of artesunate on HBSM cells and rat lungs. Western blot analysis showed that CS significantly decreased PPAR-γ expression in both HBSM cells and rat lungs, and this effect was reversed by artesunate treatment in a dose-dependent manner (Fig. 5a, b). CS can trigger TGF-β1 release, activating its downstream signalling pathway Smad2/3 cascade to promote airway remodelling. We subsequently validated the effects of artesunate on CS-induced activation of TGF-β1 signalling, and we found that TGF-β1 was significantly upregulated in CS-exposed HBSM cells and rat lungs, and these effects were reversed by artesunate treatment (Fig. 5c, f). Additionally, CSE-induced Smad2/3 phosphorylation was also inhibited by artesunate treatment (Fig. 5d, e). Taken together, these results demonstrated the attenuating effects of artesunate on both PPAR-γ upregulation and TGF-β1/Smad2/3 dephosphorylation in CS-induced airway inflammation and remodelling.

### Role of artesunate in suppressing cell proliferation by targeting PPAR-γ/TGF-β1/Smad2/3 signalling

**Fig. 6:**
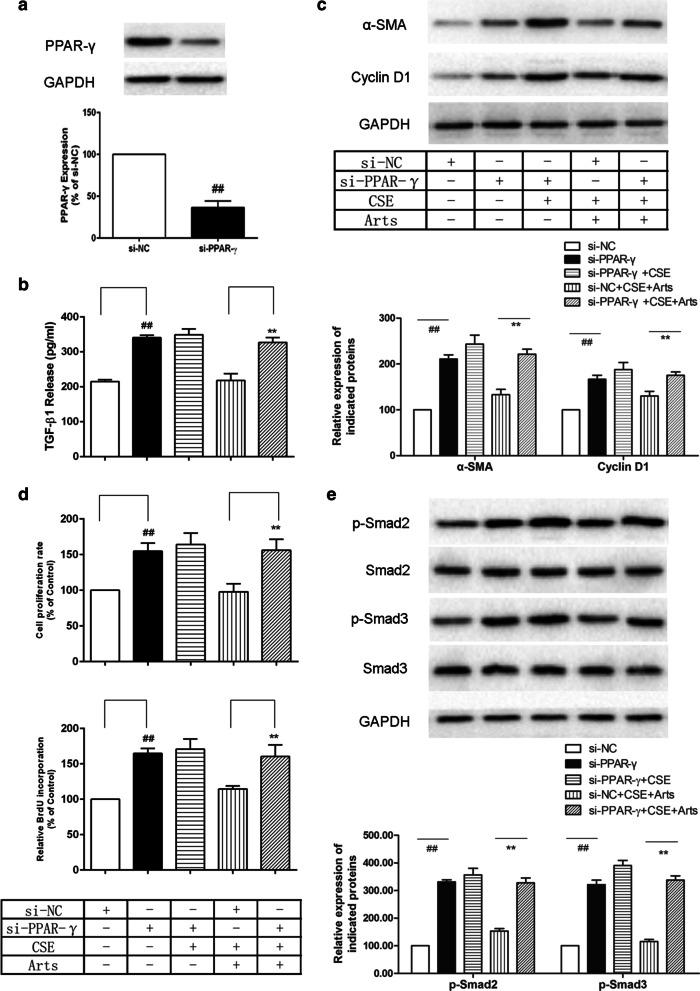
Suppressing cell proliferation by artesunate through PPAR-γ/TGF-β1/Smad2/3 signalling pathway. HBSM cells were transfected with siRNA-PPAR-γ in the presence or absence of CSE/artesunate. **a** The interference efficiency was determined by Western blot. **b** The level of TGF-β1 in supernatant of HBSM cells was determined using ELISA test. **c** The expressions of α-SMA, cyclin D1 and GAPDH were determined using Western blot. **d** The cell viability was measured by CCK-8 assay and the cell proliferation was measured by BrdU incorporation assay. **e** The expressions of p-Smad2/3, Smad2/3 and GAPDH were determined using Western blot. Data were expressed as mean ± SEM, n = 3. ^##^P < 0.01 compared to si-NC group using unpaired *t*-test; **P < 0.01 compared to si-NC + CSE + Arts group. *Arts* Artesunate

Given that the involvement of PPAR-γ and TGF-β1/Smad2/3 signalling in CSE-induced airway remodelling and the abnormal proliferation of airway smooth muscle cells directly contributes to airway remodelling [26], we investigated whether the suppressive effects of artesunate on HBSM cell proliferation were associated with PPAR-γ activation and the TGF-β1 signalling pathway. Western blot analysis showed that siRNA-PPAR-γ transfection significantly abolished PPAR-γ expression in HBSM cells (Fig. 6a). We also showed that PPAR-γ knockdown abolished the effect of articulate, as evidenced by the enhanced TGF-β1 levels in the supernatant of HBSM cells (Fig. 6b) and Smad2/3 dephosphorylation (Fig. 6e) in HBSM cells stimulated by CSE. As expected, PPAR-γ knockdown also enhanced the expression of α-SMA and cyclin D1 (Fig. 6c) and significantly increased the proliferation of CSE-exposed HBSM cells, and these effects were inhibited by artesunate, as measured by CCK-8 and BrdU assays (Fig. 6d). These results suggest that artesunate suppresses cell proliferation through the TGF-β1/Smad2/3 signalling pathway by targeting PPAR-γ.

## Discussion

The main findings of the present study are that artesunate significantly suppressed CS-induced airway inflammation as well as airway remodelling in vivo and in vitro. The effect of artesunate was associated with CS-induced airway remodelling by targeting PPAR-γ/TGF-β1/Smad2/3 signalling.

Experimental studies have already confirmed that CS exposure directly contributes to the inflammation- and oxidative stress-induced changes in structural cells seen in the lung tissue and small airways [27]. Thus, to some extent, inhibiting the inflammatory response, oxidative stress, and airway remodelling may offer viable strategies for COPD therapy. First, we established a 12-week CS-exposed rat model to evaluate the therapeutic effects of artesunate, and we found that artesunate treatment dose-dependently reduced the levels of IL-6, IL-8, TNF-α, ICAM-1, ROS and GSH in the BALF of CS-exposed rats. These results were consistent with those of another study showing that artesunate could modulate multiple inflammatory and oxidative stress mediators in a CS- or ovalbumin-exposed mouse model [28].

Previous studies in both animals and humans have shown that CS induces the production of proinflammatory cytokines such as IL-6, IL-8 and TNF-α, as well as ICAM-1, ROS and GSH, which amplify the inflammatory process and play integral roles in the coordination and persistence of inflammationin airway remodelling of COPD patients [29, 30]. These pathological changes in CS-exposed lungs were significantly ameliorated by artesunate treatment. Importantly, the increase in isometric force and decrease in lung function, including PIF, PEF, IP and IP-slope, which represent airway hyperresponsiveness and small airway resistance, respectively, were also ameliorated. In addition to its antimalarial properties, artesunate has also been reported to exhibit a variety of pharmacological activities. For example, artesunate was reported to reduce lung damage in CS-induced mice [31], which was consistent with our results. Artesunate ameliorated CS-induced airway inflammation, inhibited the PI3Kδ/Akt pathway, and restored HDAC2 activity, consequently reversing CSE-induced glucocorticoid insensitivity [28]. These findings prompted us to further characterize the underlying mechanisms in detail. Therefore, as a multitarget drug, artesunate is an example of repurposing an ‘old’ drug to treat airway remodelling, which is a key feature of COPD and asthma [32]. To further validate the animal experiment results, cellular experiments were performed on HBSM cells. It is widely accepted that CSE has a significant effect on abnormal airway contractility and the proliferation of HBSM cells [33]. Our results were consistent with these findings and further demonstrated that artesunate inhibited HBSM cell proliferation and reduced the levels of the proliferation markers cyclin D1 and the myofibroblast marker α-SMA in vivo and in vitro. However, previous studies have reported that artesunate suppresses the proliferation of human leukaemic cells by regulating c-Myb and cyclin D2 expression [34]. Moreover, artesunate significantly inhibited the proliferation of hepatoma cell lines via STAT3 inhibition and DR4 augmentation [35]. In our study, we observed that the increase in HBSM cell proliferation caused by CSE could be inhibited by artesunate, accompanied by the recovery of cyclin D1 and α-SMA expression, revealing the different mechanisms by which artesunate inhibits cell proliferation in various systems.

It has been reported that PPAR-γ activation reduces lipopolysaccharide-induced inflammation in a mouse model, suggesting that an activator of PPAR-γ may have a beneficial effect on the inflammatory response in COPD [36]. In fact, several studieshave shown reductions in the level and activity of PPAR-γ in the lungs of CS-exposed mice, smokers and COPD patients [37, 38]. Consistently, our data demonstrated that PPAR-γ expression in both rat lungs and HBSM cells was significantly inhibited in response to CS, which is in consistent with previous reports that CS could inhibit PPAR-γ activation [39]. Treatment with artesunate significantly enhanced PPAR-γ activation in vivo and in vitro, which is in consistent with previous reports that PPAR-γ agonists reversed CS-induced airway injury in bronchial epithelial cells [40]. Thus, we hypothesize that PPAR-γ activation may be involved in the protection against CS-induced airway inflammation and remodelling.

It has been reported that the TGF-β1-Smad signalling pathway can activated by CS in bronchial rat explantsand is identified as the key signalling pathway in EMT and airway remodelling [11, 41]. It has been reported that the TGF-β1/Smad2 pathway is significantly activated in bronchial smooth muscle cells exposed to CS [42]. More importantly, inhibition of the TGF-β1 gene by PPAR-γ activation can be used to treat TGF-β1-induced pathophysiological disorders such as fibrosis [43]. We further investigated whether the effect of artesunate involved the TGF-β1 signalling via the activation of PPAR-γ. We found that PPAR-γ knockdown and inactivation of TGF-β1/Smad signalling pathway attenuated the effect of artesunate on CS-induced cell proliferation in vitro, which was consistent with a previous study on the activation of PPAR-γ in various cells and diseases [44]. For instance, activation of PPAR-γ in myeloid cells could promote the progression of epithelial lung tumours by regulating the TGF-β1 signalling pathway. PPAR-γ expression was increased in NSCLC cell lines, and knockdown of PPAR-γ inhibited EMT [45]. Therefore, our results further confirmed that artesunate ameliorated CS-induced bronchial remodelling via the PPAR-γ/TGF-β1/Smad2/3 signalling pathway.

However, there are still some limitations in our study. For example, we did not carry out therapeutic medication study of artesunate in animal experiment to further prove its therapeutic effect on cigarette smoke-induced airway remodelling. In addition, our study did not completely clarify whether artesunate targeted PPAR-γ or not and whether PPAR-γ is the only target of artesunate, which will be the focus in our follow-up research.

## Conclusion

In conclusion, our results revealed that artesunate treatment significantly protected against CS-induced airway inflammation, as well as airway remodelling via PPAR-γ/TGF-β1/Smad2/3 signalling in vivo and in vitro, and provides a novel use for an ‘old’ drug to treat airway remodelling in COPD.

## Supplementary Information

**Additional file 1:** Correlation analysis of lung tissues proliferation and CyclinD1.

## Data Availability

The software and all relevant raw data are freely available to scientists.

## Abbreviations

ANOVA: One-way analysis of variance
α-SMA: Alpha-smooth muscle actin
BALF: Bronchoalveolar lavage fluid
CCh: Carbachol
CCK-8: Cell Counting Kit-8
COPD: Chronic obstructive pulmonary disease
CS: Cigarette smoke
CSE: Cigarette smoke extract
ELISA: Enzyme-linked immunosorbent assay
EMT: Epithelial–mesenchymal transition
GSH: Reduced glutathione
HBSM: Human bronchial smooth muscle
HDAC2: Histone deacetylase 2
ICAM-1: Intercellular adhesion molecule 1
IL: Interleukin
IP: Airway inside pressure
IP-slope: Airway pressure maximum rising slope
PPAR-γ: Peroxisome proliferator-activated receptor γ
ROS: Reactive oxygen species
SD: Sprague-Dawley
TGF-β1: Transforming growth factor beta 1
TNF-α: Tumor necrosis factor alpha
PIF: Peak inspiratory flow
PEF: Peak expiratory flow

## References

[1] Jones RL, Noble PB, Elliot JG, James AL (2016). Airway remodelling in COPD: it's not asthma!. Respirology.

[2] Marchini G, Carnevali S, Facchinetti F (2019). Formoterol counteracts the inhibitory effect of cigarette smoke on glucocorticoid-induced leucine zipper (GILZ) transactivation in human bronchial smooth muscle cells. Eur J Pharmacol.

[3] Chen L, Ge Q, Tjin G, Alkhouri H, Deng LH, Brandsma CA (2014). Effects of cigarette smoke extract on human airway smooth muscle cells in COPD. Eur Respir J.

[4] Knobloch J, Wahl C, Feldmann M, Jungck D, Strauch J, Stoelben E (2014). Resveratrol attenuates the release of inflammatory cytokines from human bronchial smooth muscle cells exposed to lipoteichoic acid in chronic obstructive pulmonary disease. Basic Clin Pharmacol Toxicol.

[5] Xu GN, Yang K, Xu ZP, Zhu L, Hou LN, Qi H (2012). Protective effects of anisodamine on cigarette smoke extract-induced airway smooth muscle cell proliferation and tracheal contractility. Toxicol Appl Pharmacol.

[6] Liu L, Pan Y, Zhai C, Zhu Y, Ke R, Shi W (2018). Activation of peroxisome proliferation-activated receptor-γ inhibits transforming growth factor-β1-induced airway smooth muscle cell proliferation by suppressing Smad-miR-21 signalling. J Cell Physiol.

[7] Patel HJ, Belvisi MG, Bishop-Bailey D, Yacoub MH, Mitchell JA (2003). Activation of peroxisome proliferator-activated receptors in human airway smooth muscle cells has a superior anti-inflammatory profile to corticosteroids: relevance for chronic obstructive pulmonary disease therapy. J Immunol.

[8] Ward JE, Gould H, Harris T, Bonacci JV, Stewart AG (2004). PPAR gamma ligands, 15-deoxy-delta12,14-prostaglandin J2 and rosiglitazone regulate human cultured airway smooth muscle proliferation through different mechanisms. Br J Pharmacol.

[9] Lakshmi SP, Reddy AT, Zhang Y, Sciurba FC, Mallampalli RK, Duncan SR (2014). Down-regulated peroxisome proliferator-activated receptor γ (PPARγ) in lung epithelial cells promotes a PPARγ agonist-reversible proinflammatory phenotype in chronic obstructive pulmonary disease (COPD). J Biol Chem.

[10] Zhao C, Chen W, Yang L, Chen L, Stimpson SA, Diehl AM (2006). PPARgamma agonists prevent TGFbeta1/Smad3-signalling in human hepatic stellate cells. Biochem Biophys Res Commun.

[11] Guan S, Xu W, Han F, Gu W, Song L, Ye WJ (2017). Ginsenoside Rg1 attenuates cigarette smoke-induced pulmonary epithelial-mesenchymal transition via inhibition of the TGF-β1/Smad pathway. Biomed Res Int.

[12] Pires N, Pinto P, Marçal N, Ferreira AJ, Rodrigues C, Bárbara C (2019). Pharmacological treatment of COPD - New evidence. Pulmonology.

[13] Pushpakom S, Iorio F, Eyers PA, Escott KJ, Hopper S, Wells A (2019). Drug repurposing: progress, challenges and recommendations. Nat Rev Drug Discov.

[14] Gordi T, Lepist EI (2004). Artemisinin derivatives: toxic for laboratory animals, safe for humans?. Toxicol Lett.

[15] Zhao D, Zhang J, Xu G, Wang Q (2017). Artesunate protects LPS-induced acute lung injury by inhibiting TLR4 expression and inducing Nrf2 activation. Inflammation.

[16] Verma S, Kumar VL (2018). Artesunate affords protection against aspirin-induced gastric injury by targeting oxidative stress and proinflammatory signalling. Pharmacol Rep.

[17] Ho WE, Cheng C, Peh HY, Xu FG, Tannenbaum SR, Ong CN (2012). Anti-malarial drug artesunate ameliorates oxidative lung damage in experimental allergic asthma. Free Radic Biol Med.

[18] Ng DS, Liao W, Tan WS, Chan TK, Loh XY, Wong WS (2014). Anti-malarial drug artesunate protects against cigarette smoke-induced lung injury in mice. Phytomedicine.

[19] Tan SS, Ong B, Cheng C, Ho WE, Tam JKC, Stewart AG (2014). The antimalarial drug artesunate inhibits primary human cultured airway smooth muscle cell proliferation. Am J Respir Cell Mol Biol.

[20] Wang Y, Wang A, Zhang M, Zeng HL, Lu Y, Liu L (2019). Artesunate attenuates airway resistance in vivo and relaxes airway smooth muscle cells in vitro via bitter taste receptor-dependent calcium signalling. Exp Physiol.

[21] Wang C, Xuan X, Yao W, Huang G, Jin J (2015). Anti-profibrotic effects of artesunate on bleomycin-induced pulmonary fibrosis in Sprague Dawley rats. Mol Med Rep.

[22] Song Y, Lu HZ, Xu JR, Wang XL, Zhou W, Hou LN (2015). Carbocysteine restores steroid sensitivity by targeting histone deacetylase 2 in a thiol/GSH-dependent manner. Pharmacol Res.

[23] Choudhury G, MacNee W (2017). Role of inflammation and oxidative stress in the pathology of ageing in COPD: potential therapeutic interventions. COPD.

[24] Wiegman CH, Michaeloudes C, Haji G, Narang P, Clarke CJ, Russell KE (2015). Oxidative stress-induced mitochondrial dysfunction drives inflammation and airway smooth muscle remodelling in patients with chronic obstructive pulmonary disease. J Allergy Clin Immunol.

[25] Tejwani V, Yun X, Sikka G, Shimoda L, Suresh K (2019). Airway epithelial genomic signatures in steroid-resistant COPD; role for SMAD3 in vascular remodelling in pulmonary hypertension; regulation of lung endothelial cell function by VEGFR3. Am J Respir Cell Mol Biol.

[26] Wang L, Feng X, Hu B, Xia Q, Ni X, Song Y (2018). P2X4R promotes airway remodelling by acting on the phenotype switching of bronchial smooth muscle cells in rats. Purinergic Signal.

[27] Laucho-Contreras ME, Taylor KL, Mahadeva R, Boukedes SS, Owen CA (2015). Automated measurement of pulmonary emphysema and small airway remodelling in cigarette smoke-exposed mice. J Vis Exp.

[28] Luo Q, Lin J, Zhang L, Li H, Pan L (2015). The anti-malaria drug artesunate inhibits cigarette smoke and ovalbumin concurrent exposure-induced airway inflammation and might reverse glucocorticoid insensitivity. Int Immunopharmacol.

[29] Zhang J, Liu Y, Shi J, Larson DF, Watson RR (2002). Side-stream cigarette smoke induces dose-response in systemic inflammatory cytokine production and oxidative stress. Exp Biol Med (Maywood).

[30] Son ES, Park JW, Kim YJ, Jeong SH, Hong JH, Kim SH (2020). Effects of antioxidants on oxidative stress and inflammatory responses of human bronchial epithelial cells exposed to particulate matter and cigarette smoke extract. Toxicol In Vitro.

[31] Luo QZ, Lin JT, Li H, Pan L (2016). Effects of artesunate on cigarette smoke-induced lung oxidative damage in mice and the expression of Nrf2 and the possible mechanism. Zhonghua Yi Xue Za Zhi.

[32] Joubert P, Hamid Q (2005). Role of airway smooth muscle in airway remodelling. J Allergy Clin Immunol.

[33] Guan P, Cai W, Yu HP, Wu ZY, Li W, Wu J (2017). Cigarette smoke extract promotes proliferation of airway smooth muscle cells through suppressing C/EBP-α expression. Exp Ther Med.

[34] Chen S, Gan S, Han LJ, Li X, Xie XQ, Zou DB, Sun H (2020). Artesunate induces apoptosis and inhibits the proliferation, stemness, and tumorigenesis of leukemia. Ann Transl Med.

[35] Ilamathi M, Sivaramakrishnan V (2017). Artesunate acts as fuel to fire in sensitizing HepG2 cells towards TRAIL mediated apoptosis via STAT3 inhibition and DR4 augmentation. Biomed Pharmacother.

[36] Lin MH, Chen MC, Chen TH, Chang HY, Chou TC (2015). Magnolol ameliorates lipopolysaccharide-induced acute lung injury in rats through PPAR-γ-dependent inhibition of NF-kB activation. Int Immunopharmacol.

[37] Simon L, Jonathan P, Hannah M, Spicer D, Woodman P, Fox JC (2014). The effect of peroxisome proliferator-activated receptor-γ ligands on in vitro and in vivo models of COPD. Eur Respir J.

[38] Spears M, McSharry C, Thomson NC (2006). Peroxisome proliferator-activated receptor-gamma agonists as potential anti-inflammatory agents in asthma and chronic obstructive pulmonary disease. Clin Exp Allergy.

[39] Yin Y, Hou G, Li E, Wang Q, Kang J (2014). PPAR-γ agonists regulate tobacco smoke-induced Toll like receptor 4 expression in alveolar macrophages. Respir Res.

[40] Yin Y, Hou G, Li ER, Wang QY, Kang J (2013). Regulation of cigarette smoke-induced toll-like receptor 4 expression by peroxisome proliferator-activated receptor-gamma agonists in bronchial epithelial cells. Respirology.

[41] Sagara H, Okada T, Okumura K, Ogawa H, Ra C, Fukuda T, Nakao A (2002). Activation of TGF-beta/Smad2 signalling is associated with airway remodelling in asthma. J Allergy Clin Immunol.

[42] Wang RD, Wright JL, Churg A (2005). Transforming growth factor-beta1 drives airway remodelling in cigarette smoke-exposed tracheal explants. Am J Respir Cell Mol Biol.

[43] Lee CH, Kim HD, Shin SM, Kim SG (2008). A novel mechanism of PPARgamma regulation of TGFbeta1: implication in cancer biology. PPAR Res.

[44] Wang S, Dougherty EJ, Danner RL (2016). PPARγ signalling and emerging opportunities for improved therapeutics. Pharmacol Res.

[45] Sippel TR, Johnson AM, Li HY, Hanson D, Nguyen TT, Bullock BL (2019). Activation of PPARγ in myeloid cells promotes progression of epithelial lung tumors through TGFβ1. Mol Cancer Res.

