# Supplementary figures and images for: Artesunate ameliorates cigarette smoke-induced airway remodelling via PPAR-γ/TGF-β1/Smad2/3 signalling pathway
Source: Respir Res. 2021 Mar 23;22:91. doi: 10.1186/s12931-021-01687-y (PMC7989207; doi:10.1186/s12931-021-01687-y)

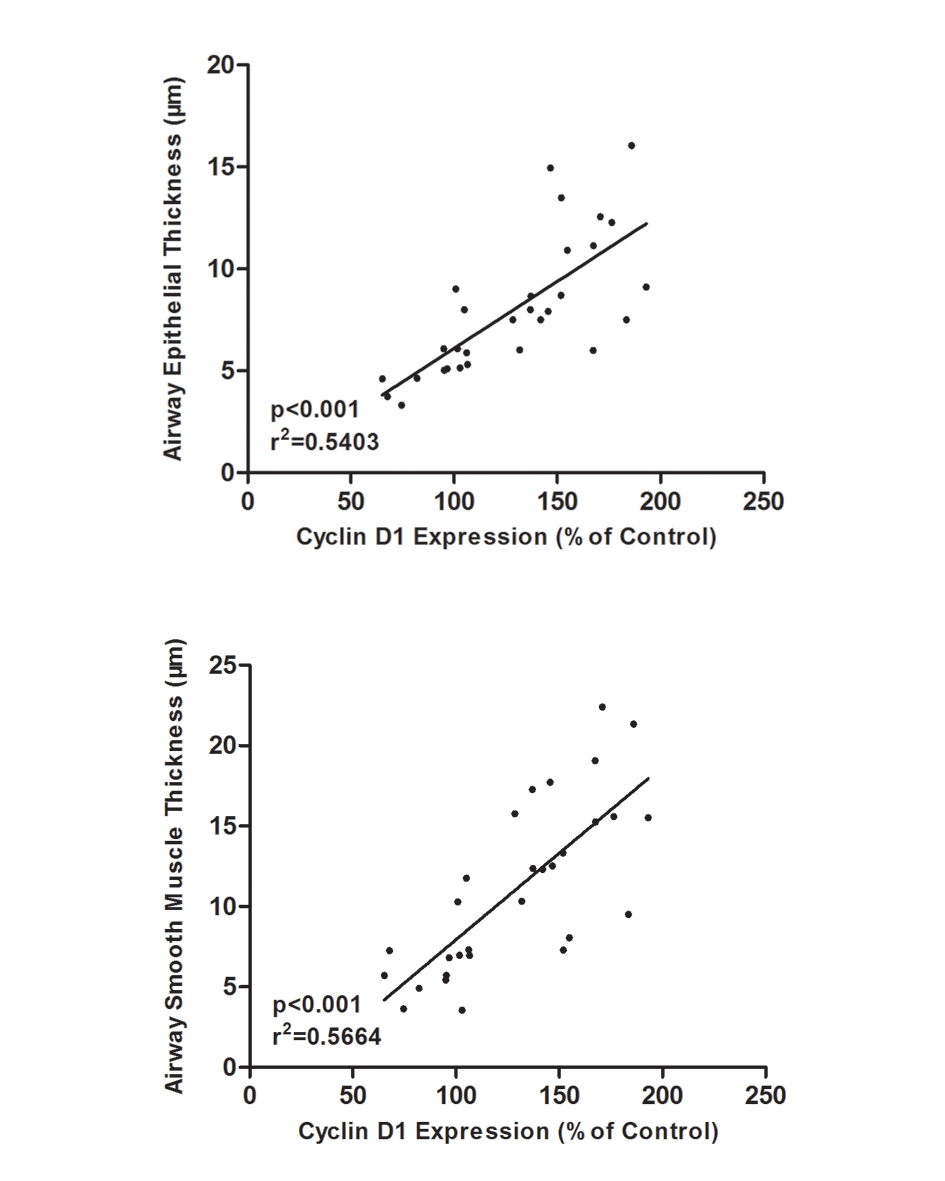

Supplement: Supplementary file 1 — Additional file 1: Correlation analysis of lung tissues proliferation and CyclinD1. [file 12931_2021_1687_MOESM1_ESM.tif]
